# Prevalence of Sexually Transmitted Viral and Bacterial Infections in HIV-Positive and HIV-Negative Men Who Have Sex with Men in Toronto

**DOI:** 10.1371/journal.pone.0158090

**Published:** 2016-07-08

**Authors:** Robert S. Remis, Juan Liu, Mona R. Loutfy, Wangari Tharao, Anuradha Rebbapragada, Sanja Huibner, Maya Kesler, Roberta Halpenny, Troy Grennan, Jason Brunetta, Graham Smith, Tatjana Reko, Rupert Kaul

**Affiliations:** 1 Dalla Lana School of Public Health, University of Toronto, Toronto, Ontario, Canada; 2 Women’s College Research Institute, Women’s College Hospital, University of Toronto, Toronto, Ontario, Canada; 3 Maple Leaf Medical Clinic, Toronto, Ontario, Canada; 4 Women’s Health in Women’s Hands Community Health Centre, Toronto, Ontario, Canada; 5 Public Health Laboratory–Toronto Public Health Ontario, Toronto, Ontario, Canada; 6 Department of Laboratory Medicine and Pathobiology, University of Toronto, Toronto, Ontario, Canada; 7 Department of Medicine, University Health Network, University of Toronto, Toronto, Ontario, Canada; 8 Department of Medicine, McMaster University, Hamilton, Ontario, Canada; Johns Hopkins School of Public Health, UNITED STATES

## Abstract

**Background:**

Hepatitis B (HBV), hepatitis C (HCV) and other sexually transmitted infections (STIs) have been associated with HIV transmission risk and disease progression among gay men and other men who have sex with men (MSM), but the frequency and distribution of STIs in this community in Canada has not been extensively studied.

**Methods:**

We recruited MSM living with and without HIV from a large primary care clinic in Toronto. Participants completed a detailed socio-behavioural questionnaire using ACASI and provided blood for syphilis, HIV, HBV and HCV, herpes simplex virus type 1 (HSV-1) and type 2 (HSV-2), and human cytomegalovirus (CMV) serology, urine for chlamydia and gonorrhea, and a self-collected anal swab for human papillomavirus (HPV) molecular diagnostics. Prevalences were expressed as a proportion and compared using chi-square.

**Results:**

442 MSM were recruited, 294 living with HIV and 148 without. Active syphilis (11.0% vs. 3.4%), ever HBV (49.4% vs. 19.1%), HCV (10.4% vs. 3.4%), HSV-2 (55.9% vs. 38.2%), CMV (98.3% vs. 80.3%) and high-risk (HR) anal HPV (67.6% vs. 51.7%) infections were significantly more common in men living with HIV. Chlamydia and gonorrhea were infrequent in both groups. Regardless of HIV infection status, age and number of lifetime male sexual partners were associated with HBV infection and lifetime injection drug use with HCV infection.

**Conclusions:**

Syphilis and viral infections, including HBV, HCV, HSV-2, CMV, and HR-HPV, were common in this clinic-based population of MSM in Toronto and more frequent among MSM living with HIV. This argues for the implementation of routine screening, vaccine-based prevention, and education programs in this high-risk population.

## Introduction

Despite significant advances in the care of those affected by the human immunodeficiency virus (HIV) over the last several decades, this infection remains a substantial public health challenge. In Canada, the HIV epidemic has disproportionately impacted several communities, specifically gay men, bisexual men, and other men who have sex with men (MSM), people who inject drugs (IDU), persons from HIV-endemic countries and Aboriginal people[1]. The estimated HIV prevalence in Canada in 2011 was 208.0 per 100,000 population, and nearly half (49.7%) of those living with HIV were MSM[1]. Furthermore, 15% of the approximately 108,000 MSM living in Ontario are estimated to be living with HIV[2]. This proportion is higher in the large urban centres, with the prevalence among Toronto MSM estimated to be 18%[2].

MSM and bisexual men are also at greater risk for other sexually transmitted infections (STIs) compared to heterosexual populations, including *Neisseria gonorrhoeae*, *Chlamydia trachomatis*, *Treponema pallidum*, herpes simplex virus type 1 (HSV-1) and type 2 (HSV-2), human cytomegalovirus (CMV), and human papillomavirus (HPV)[3–8]. In addition to their direct clinical effects, these STIs may cause anogenital ulcers or mucosal inflammation, potentiating the transmission of HIV[9–15]. Furthermore, studies have found that HIV-infected MSM have a higher prevalence of co-infection with other STIs than HIV-negative MSM[5, 10, 16–18].

Hepatitis B virus (HBV), hepatitis C (HCV) and HIV are all blood-borne infections and share common routes of transmission, so co-infection by these pathogens is common[19]. While HIV increases the risk of HBV and HCV-related liver disease progression[20], the effect of HBV and HCV on HIV disease progression is unclear[21]. Nonetheless, there is evidence that the presence of HBV or HCV is independently associated with an increased risk of progression to AIDS and death[22–25]. HBV is transmitted through perinatal, percutaneous, or sexual contact with infectious biologic fluids. Canada is a region of low endemicity of HBV infection; about 5% of Canadians have had hepatitis B at some time in their lives, and 0.7–0.9% are chronically infected[26]. However, certain vulnerable populations are disproportionately affected, including MSM, Aboriginal peoples, street-involved youth, and people with current or prior history of incarceration. The most commonly identified risk factors are condomless sex and injection drug use (IDU)[26]. The estimated worldwide prevalence of HCV infection is 2.8%[27]. HCV is most efficiently spread through exposure to contaminated blood and blood products, particularly in persons with IDU[19]. Sexual transmission of HCV is inefficient and uncommon in the general population; however, it is becoming widely recognized as a growing public health issue among people living with HIV. Studies indicate that MSM who are living with HIV and who practice condomless sex are at increased risk for sexually acquired HCV[28–30].

The epidemiology of these co-infections among both HIV-positive and HIV-negative MSM is not well characterized in Canada. Additionally, MSM in Toronto are significantly affected by the HIV epidemic, with an estimated HIV prevalence of 18% and an annual incidence of 1%[2]. Therefore, data regarding the prevalence and correlates of these co-infections in HIV-infected and uninfected men will inform the local clinicians, public health staff and community organizations that provide screening, care and counseling to the MSM community in Toronto. We aimed to determine the prevalence and correlates of bacterial and viral STIs among MSM living with and without HIV in Toronto, Ontario.

## Methods

### Participants and recruitment

The study population consisted of MSM who were 18 years of age or older and living in Greater Toronto. Subjects were recruited from Maple Leaf Medical Clinic (MLMC) from September 2010 to June 2012. This clinic is located in downtown Toronto and predominantly serves a large MSM community with a high HIV prevalence. All participants provided informed written consent and the study protocol was approved by the HIV Research Ethics Board of the University of Toronto. The study utilized a non-random convenience, non-representative sampling approach with deliberate over-sampling of HIV-infected men. A list of randomly selected eligible clinic subjects was prepared, and those with a scheduled appointment were invited to speak to the Research Coordinator if interested in participation.

### Study procedures

Participants completed a self-administered questionnaire using ACASI (Audio Computer Assisted Self-Interview), (Questionnaire Development System (QDS) Version 2.5, Nova Research Company, Bethesda, Maryland, USA) that included demographic information, sexual behaviour, history of STIs and other medical conditions.

Participants then provided a first-void urine specimen for *Neisseria gonorrhoea* and *Chlamydia trachomatis* molecular diagnostics, and self-collected an anal swab for HPV molecular diagnostics. Pre-test counselling for HIV and STI testing was performed, and blood collected for HIV, HSV-1, HSV-2, CMV, syphilis, HBV, and HCV serology.

### Laboratory methods

*Neisseria gonorrhoeae* and *Chlamydia trachomatis* were tested in first-void urine specimens by nucleic acid amplification testing (NAAT) (ProbeTec™ ET Amplified DNA Assay, Becton Dickinson, Franklin Lakes, NJ, USA). The chemiluminescent microparticle immunoassay (CMIA) (Abbott Laboratories, Abbott Park, IL, USA) was used for syphilis screening and, if positive, the rapid plasma reagin (RPR, Pulse Diagnostic Inc, Burlington, ON) and confirmatory *Treponema pallidum* particle agglutination (TPPA) assay (Serodia TPPA, Fujirebo Inc) were performed. Participants were classified as having active syphilis infection if the CMIA, TPPA and RPR were all reactive, or as having treated syphilis if CMIA and TPPA were reactive and RPR was non-reactive. Participants self-reporting prior syphilis were classified as having treated syphilis, regardless of syphilis CMIA.

HIV testing was performed on serum by enzyme immunoassay (EIA; AxSYM HIV 1/2 gO, Abbott Diagnostics Division, Wiesbaden, Germany). If reactive, the EIA was repeated and confirmed at the Ontario Public Health Laboratory by combination antigen (Ag)/antibody (Ab) testing (Architect Ag/Ab Combo, Abbott Diagnostics, Abbott Park, IL, USA) and by Western blot. Serologic testing was also performed for herpes simplex, types 1 and 2 (Herpes Simplex Virus ELISA IgG, Focus Diagnostics, Cypress, CA, USA); human cytomegalovirus (CMV; AxSYM CMV IgG; Abbott Diagnostics Division, Wiesbaden, Germany); HCV antibody (AxSYM HCV Version 3.0), with serologic confirmation of HCV-positive tests (Bio-Rad Monolisa anti-HCV Plus Version 2, Bio-Rad Laboratories, Montreal, Quebec); HBV surface antigen (AxSYM HbSAg V2 assay, Abbott Diagnostics); and HBV surface and core antibodies (AxSYM Core 2.0, Abbott Diagnostics). Participants were considered actively infected by HBV if HBsAg was present and ever infected if HBsAg or anti-HBc or anti-HBs if not vaccinated were detected. 46 HPV genotypes including 13 high-risk (HR) HPV types (16, 18, 31, 33, 35, 39, 45, 51, 52, 56, 58, 59, 68) from self-collected anal swab sample were identified using the Luminex microsphere HPV genotyping assay as per the published protocol[31]. We considered a specimen to be positive for HPV if it was positive for any of the 46 genotypes.

### Statistical analysis

Laboratory results were double-entered in a Microsoft Excel spreadsheet. The data from the ACASI questionnaire and the laboratory results were analyzed using SAS version 9.3 (SAS, Cary, NC, USA). We examined prevalence (with exact binomial 95% confidence interval [CI]) of STIs stratified by HIV status. Based on previous published reports, for each STI we examined a set of predictors based on demographics (sex, age, ethnic group, region of birth, education level, marital status, annual household income), sexual behaviours (number of lifetime male partners, condomless anal sex with casual partner in the previous 6 months), other health behaviours (smoking, alcohol drink, lifetime injection drug use) and other STIs tested. Wilcoxon-Mann-Whitney test for continuous variables and chi-square test for categorical variables were used for inter-group comparison. Factors with significance level of p<0.10 in the bivariate analysis were included and remained in multivariate logistic regression model with stepwise backwards elimination. Adjusted odds ratio (AOR) and its 95% CI were estimated. Values of p less than 0.05 (two-tailed) were considered statistically significant. Since the focus was the individual null hypotheses of each outcome, the Bonferroni correction for multiple comparisons was not applied, due to concerns over the risk of increased Type II error[32].

## Results

### Study population

We deliberately oversampled HIV-infected participants, based on prior HIV status, and demographic characteristics are shown in Table 1. The analysis included 294 MSM living with HIV and 148 without, with a median age of 45 years (interquartile range [IQR], 38–50) and 44 years (IQR, 37–50), respectively; the youngest participant was 22 years. Compared to HIV-uninfected participants, MSM living with HIV were more likely to be single and to report ethnicity as other than white, and reported lower levels of education and annual household income.

**Table 1:** Demographic characteristics among MSM living with and without HIV in Toronto.

		Living with HIV	Without HIV	p value
Total participants		294	148	
Age (years)				
	Mean	44.4	44.3	NS
	Median (IQR)	45 (38–50)	44 (37–50)	
Marital status				
	Married/common-law (female)	2 (0.7%)	1 (0.7%)	0.0013
	Married/common-law (male)	56 (19.0%)	55 (37.4%)	
	Separated/divorced/widowed (female)	14 (4.8%)	6 (4.1%)	
	Separated/divorced/widowed (male)	41 (13.9%)	18 (12.2%)	
	Single	181 (61.6%)	67 (45.6%)	
Education				
	Elementary school	4 (1.4%)	0 (0.0%)	0.0001
	Secondary school	62 (21.1%)	11 (7.4%)	
	Some college/university	79 (26.9%)	34 (23.0%)	
	Completed college/university	117 (39.8%)	71 (48.0%)	
	Some/completed graduate education	32 (10.9%)	32 (21.6%)	
Annual household income				
	< $20,000	121 (42.6%)	29 (20.4%)	<0.0001
	$20,000–$39,999	48 (16.9%)	15 (10.6%)	
	$40,000–$59,999	46 (16.2%)	22 (15.5%)	
	$60,000–$79,999	17 (6.0%)	22 (15.5%)	
	$80,000 +	52 (18.3%)	54 (38.0%)	
Region of birth				
	Canada	221 (75.2%)	113 (76.4%)	NS
	United States	5 (1.7%)	0 (0.0%)	
	Central/South America/Caribbean	37 (12.6%)	14 (9.5%)	
	Europe	18 (6.1%)	11 (7.4%)	
	Other	11 (4.1%)	10 (6.8%)	
Current immigration status				
	Canadian citizen	275 (93.5%)	139 (93.9%)	NS
	Landed/permanent resident	13 (4.4%)	5 (3.4%)	
	Refugee/refugee claimant	6 (2.0%)	2 (1.4%)	
	Other	0 (0.0%)	2 (1.4%)	
Language 1^st^ learned and still understand				
	English	246 (84.8%)	126 (86.3%)	NS
	French	10 (3.4%)	3 (2.1%)	
	Other	34 (11.7%)	17 (11.6%)	
Race/ethnicity				
	White	211 (75.1%)	119 (82.6%)	0.011
	Black	20 (7.1%)	3 (2.1%)	
	Asian	9 (3.2%)	10 (6.9%)	
	Latin American	14 (5.0%)	7 (4.9%)	
	Aboriginal	17 (6.0%)	1 (0.7%)	
	Mixed	10 (3.6%)	4 (2.8%)	

The majority of subjects identified themselves as gay/homosexual men, although 6.8% (20/294) of participants living with HIV and 5.4% (8/148) without reported being bisexual. MSM living with HIV had more lifetime male partners than HIV-negative men, with 82.0% and 72.8% respectively reporting more than 50 lifetime partners. However, HIV-uninfected men were currently more sexually active and reported more male partners than their counterparts living with HIV, with a median of 5 (IQR, 1–15) and 3 (IQR, 1–8) male partners in the previous 6 months; 62.9% (185/294) of those living with HIV and 66.2% (98/148) of those without reported sex with a casual male partner in the previous 6 months, and the proportion reporting condomless anal sex with casual partners in the previous 6 months was 42.5% (125/294) and 27.0% (40/148), respectively.

### Co-infections

We next assessed the prevalence of bacterial and viral pathogens (Table 2), and examined covariates of reportable infections where prevalence was found to be above 10% in either infected or uninfected MSM participants. 49.4% of MSM living with HIV had serologic evidence of lifetime HBV infection versus 19.1% of those without. Active HBV infection tended to be more common in men living with HIV, although this difference was not statistically significant (2.7% vs. 0.7%). While HBV vaccination was frequent, regardless of HIV status (83.3% and 87.4% for men living with and without HIV, respectively), over half of men living with HIV (103/182) and those without (50/90) had received only one or two of the three recommended doses where data were available. HCV infection was found among 10.4% of MSM living with HIV and 3.4% of those without HIV (p = 0.02); however, this difference was largely due to self-reported IDU (any lifetime IDU). The prevalence of HCV was 36.1% among MSM living with HIV who reported prior use of intravenous drugs (IDU) and 37.5% among HIV-uninfected MSM-IDUs, versus 3.6% in MSM living with HIV who reported no prior IDU and 1.4% among HIV-uninfected MSM without prior IDU.

**Table 2:** Prevalence of bacterial and viral pathogens among MSM living with and without HIV in Toronto*.

	Living with HIV			Living without HIV			p value
	Tested	Positive	Prevalence % (95% CI**)	Tested	Positive	Prevalence % (95% CI**)	
Chlamydia (urethral)	290	3	1.0% (0.2–3.0%	148	0	0.0% (0.0–2.5%)	NS
Gonorrhea (urethral)	292	1	0.34% (0.0–1.9%)	147	0	0.0% (0.0–2.5%)	NS
Syphilis active	290	32	11.0% (7.7–15.2%)	147	5	3.4% (1.1–7.8%)	0.012
Syphilis ever	290	107	36.9% (31.3–42.7%)	147	30	20.4% (14.2–27.8%)	0.0007
HSV-1	288	226	78.5% (73.3–83.1%)	144	100	69.4% (61.2–76.8%)	0.040
HSV-2	288	161	55.9% (50.0–61.7%)	144	55	38.2% (30.2–46.7%)	0.0005
Cytomegalovirus	292	287	98.3% (96.1–99.4%)	147	118	80.3% (72.9–86.4%)	<0.0001
High risk HPV, anal	284	192	67.6% (61.8–73.0%	145	75	51.7% (43.3–60.1%	0.0013
HCV	289	30	10.4% (7.1–14.5%)	148	5	3.4% (1.1–7.7%)	0.018
HBV infected ^†^	291	8	2.7% (1.2–5.3%)	141	1	0.71% (0.0–3.9%)	NS
HBV ever ^‡^	291	144	49.4% (43.6–55.4%)	141	27	19.1% (13.0–26.6%)	<0.0001

* The total number of participants tested for individual STIs varied due to insufficient specimens or invalid laboratory results

** 95% exact binomial confidence interval

^†^ Infected with HBV: HBsAg, with or without other HBV markers

^‡^ Ever infected with HBV: HBsAg, anti-HBc, or anti-HBs if not vaccinated for HBV

Syphilis infection (both ever infected and active/current infection) was more prevalent among MSM living with HIV (ever infected, 36.9% vs. 20.4%, p = 0.0007; actively infected, 11.0% vs. 3.4%, p = 0.01, respectively). Urethral chlamydia and gonorrhea were infrequent. CMV infection was very common in both groups, and was detected in nearly all HIV-infected men (98.3%) compared to 80.3% in HIV-uninfected men, p<0.0001. HSV-1 infection was present among 78.5% of MSM living with HIV and 69.4% of those without (p = 0.04) and HSV-2 infection was also more frequent in HIV-infected men (55.9% vs. 38.2%; p = 0.0005). Anal HPV infection (either HR or LR HPV genotypes) was detected in 88.4% of men living with HIV and 77.9% of those without (p = 0.004). The prevalence of any HR HPV genotype infection was 67.6% and 51.7% in MSM living with and without HIV (p = 0.001).

### Covariates of viral hepatitis

Among HIV-negative MSM, factors associated with ever having HBV infection in multivariable analysis are shown in Table 3; age was of borderline significance in relation to HBV infection, while the number of lifetime partners and HBV vaccination remained significantly associated with HBV status. Among MSM living with HIV, prior HBV infection was associated with older age, ever smoking, alcohol abstinence, HSV-2, syphilis infection and HBV vaccination in both bivariate and multivariate analyses (Table 3). Among subjects with available data, we observed a dose-response effect of the number of doses of HBV vaccine on ever HBV infection regardless of HIV infection status. Among men living with HIV the proportions of ever HBV infection were 88.9% for unvaccinated participants, 58.3% for those with one dose, 39.2% with two doses, and 25.3% among fully-vaccinated men (p for trend <0.0001). Among HIV-uninfected men the proportions were 58.8%, 0.0%, 11.6% and 5.0% (p for trend < 0.0001).

**Table 3:** Correlates of hepatitis B virus infection among MSM in Toronto.

	HBV infection	Non-infection	% HBV infection	p value	Adjusted odds ratio*(95%CI)	p value
Participants living without HIV**						
Age (years)						
Mean	50.4	43.1		0.0009	1.60 (0.95–2.70)^†^	0.079
Median (IQR)	51.0 (42–55)	44.0 (35–49)				
Education						
Some college/university or less	13	29	31.0%	0.037		
Completed college/university or high	14	85	14.1%			
Annual household income						
Less than $50,000	17	36	32.1%	0.0093		
$50,000 or more	10	72	12.2%			
Number of lifetime male partners						
50+	24	80	23.1%	0.091	18.3 (1.86–180.4)	0.013
<50	3	33	8.3%		1.0	
Injection drug use (lifetime)						
Yes	4	4	50.0%	0.045		
No	23	109	17.4%			
Cytomegalovirus infection						
Yes	26	89	22.6%	0.047		
No	1	24	4.0%			
Syphilis						
Yes	11	19	36.7%	0.009		
No	15	95	13.6%			
HBV vaccination						
No	10	7	58.8%	<0.0001	3.45 (1.65–7.19)	0.0010
Yes	14	104	11.9%		1.0	
Participants living with HIV***						
Age (years)						
Mean	47.3	41.5		<0.0001	1.90 (1.35–2.67)^†^	0.0003
Median (IQR)	47.0 (43–52)	42.0 (34–47)				
Education						
Some college/university or less	80	64	55.6%	0.053		
Completed college/university or high school	64	83	43.5%			
Number of lifetime male partners						
50+	121	113	51.7%	0.11		
<50	20	32	38.5%			
Smoking						
Ever	114	92	55.3%	0.003	2.30 (1.21–4.39)	0.012
Never	30	55	35.3%		1.0	
Alcohol drinking in the previous 6 months						
No	27	13	67.5%	0.022	2.86 (1.20–6.82)	0.018
Yes	117	134	46.6%		1.0	
HSV-2 infection						
Yes	94	66	58.8%	0.0006	1.86 (1.03–3.35)	0.037
No	47	78	37.6%		1.0	
Syphilis						
Yes	64	42	60.4%	0.007	1.92 (1.04–3.56)	0.039
No	78	103	43.1%		1.0	
Hepatitis C virus infection						
Yes	20	10	66.7%	0.069		
No	121	135	47.3%			
HBV vaccination						
No	40	5	88.9%	<0.0001	2.87 (1.71–4.81)	<0.0001
Yes	88	136	39.3%		1.0	

* Logistic regression with backward elimination, p<0.10 stay in the model, initially included all variables listed in the table

**125 HIV-negative MSM with all data

*** 250 HIV-positive MSM with all data

^†^ Adjusted odds ratio in 10 years of age increase

Lifetime IDU was strongly associated with HCV infection among HIV-uninfected MSM (37.5% vs. 1.4%, p = 0.0010); no associations were seen with sexual behaviours or STIs. Table 4 summarizes the results of the analysis of the covariates of HCV infection among MSM living with HIV. Here, HSV-2 and syphilis infection were associated with HCV infection in bivariate analysis. Age, current smoking, alcohol abstinence in the previous 6 months, IDU and HBV infection remained significantly related to HCV infection in the multivariate analysis.

**Table 4:** Correlates of hepatitis C virus infection among MSM living with HIV in Toronto.

	HCV infection	Non-infection	% HCV infection	p value	Adjusted odds ratio* (95%CI)	p value
Age (years)						
Mean	46.7	44.1		0.11	1.97 (1.02–3.78)^†^	0.043
Median (IQR)	47.0 (45–52)	45.0 (38–50)				
Current smoking						
Yes	18	94	16.1%	0.020	3.9 (1.33–11.42)	0.013
No	12	165	6.8%		1.0	
Alcohol drinking in the previous 6 months						
No	10	30	25.0%	0.0032	5.83 (1.72–19.71)	0.0046
Yes	20	229	8.0%		1.0	
Injection drug use (lifetime)2						
Yes	22	39	36.1%	<0.0001	49.4 (13.7–178.4)	<0.0001
No	8	217	3.6%		1.0	
HSV-2 infection						
Yes	21	138	13.2%	0.056		
No	7	117	5.6%			
Syphilis						
Yes	17	90	15.9%	0.023		
No	12	166	6.7%			
HBV infection						
Yes	20	121	14.2%	0.069	3.19 (1.06–9.55)	0.039
No	10	135	6.9%		1.0	

* Logistic regression with backward elimination, p<0.10 stay in the model, initially included all variables listed in the table, among 272 HIV-positive MSM with all data

^†^ Adjusted odds ratio in 10 years of age increase

Note: other variables examined but not significant in the univariate analysis: education, annual household income, region of birth, marital status, race/ethnicity, number of lifetime male partners, unprotected anal sex with casual partner in the previous 6 months, cytomegalovirus and HPV infection

Among HBV-infected men living with HIV, 31.6% of were aware of their HBV infection status, and the inverse association between alcohol drinking and HBV infection persisted when these participants were excluded, implying that it was not predicated on an awareness of HBV infection. The great majority (93%) of HCV-infected men living with HCV were aware of their HCV infection, and so a similar analysis was not feasible.

### Covariates of syphilis infection

Among HIV-uninfected MSM, the lifetime prevalence of syphilis infection (“ever syphilis”) increased with increasing age (20–29 years, 0.0%; 30–54, 16.8%, and 55+, 45.5%); this was not the case among MSM living with HIV. We then assessed the correlates of ever having syphilis infection by HIV status (see Table 5). Condomless anal sex with casual partners in the previous 6 months was associated with ever syphilis regardless of HIV infection status, but in multivariate analysis, remained significant only among men living with HIV. In bivariate analysis, condomless oral sex with a HIV-infected partner (either casual or regular partner) in the previous 6 months was related to syphilis infection for both MSM living with and without HIV (36.7% vs. 17.1%, and 44.7% vs. 28.2%, respectively), but significance was lost in multivariate analysis.

**Table 5:** Correlates of ever syphilis infection among MSM in Toronto.

	Syphilis infection	Non-infection	% Syphilis infection	p value	Adjusted odds ratio* (95%CI)	p value
Participants living without HIV**						
Age (years)						
Mean	49.7	42.7		0.0018	2.61 (1.45–4.68)^†^	0.0013
Median (IQR)	49.0 (42–58)	43.5 (35–49)				
Household income						
Less than $20,000	11	18	37.9%	0.019	6.41 (1.84–22.40)	0.0036
$20,000 or more	18	94	16.1%		1.0	
UAI with casual partners in past 6 months						
Yes	12	28	30.8%	0.11		
No	18	89	16.8%			
Injection drug use						
Yes	6	2	75.0%	0.0008	51.78 (3.65–734.8)	0.0035
No	23	115	16.7%		1.0	
HSV-2 infection					3.11 (0.99–9.71)	0.051
Yes	18	36	33.3%	0.0089	1.0	
No	12	77	13.5%			
Cytomegalovirus infection						
Yes	29	88	24.8%	0.022		
No	1	28	3.4%			
HBV infection						
Yes	11	15	42.3%	0.0090		
No	19	95	16.7%			
Anal HPV infection						
Yes	28	85	24.8%	0.048	10.02 (1.05–95.48)	0.045
No	2	29	6.5%		1.0	
Participants living with HIV***						
Region of birth						
Canada/United States	74	148	33.3%	0.026	0.41 (0.20–0.84)	0.014
Other	33	34	49.3%		1.0	
Ethnicity						
White	70	137	33.8%	0.064		
Other	33	37	47.1%			
Number of lifetime male partners						
50+	94	139	40.3%	0.003	2.56 (1.04–6.34)	0.042
<50	9	43	17.3%		1.0	
UAI with casual partners in past 6 months						
Yes	59	63	48.4%	0.0009	1.89 (1.06–3.35)	0.030
No	48	120	28.6%		1.0	
Alcohol drinking in the previous 6 months						
No	21	18	53.8%	0.029		
Yes	86	165	34.3%			
Injection drug use						
Yes	35	25	53.8%	0.0002	3.18 (1.58–6.38)	0.0012
No	70	157	30.8%		1.0	
HSV-2 infection						
Yes	67	91	42.4%	0.032		
No	37	89	29.4%			
HCV infection						
Yes	17	12	58.6%	0.023		
No	90	166	35.2%			
HBV infection						
Yes	64	78	45.1%	0.0068	2.67 (1.48–4.79)	0.0011
No	42	103	29.0%		1.0	
Anal HPV infection						
Yes	100	148	40.3%	0.0089	4.11 (1.26–13.36)	0.019
No	5	28	15.2%		1.0	

* Logistic regression with backward elimination, p<0.10 stay in the model.

** 125 HIV-negative MSM with all data

*** 246 HIV-positive MSM with all data.

^†^ Adjusted odds ratio in 10 years of age increase

## Discussion

HIV disproportionately affects the MSM community in both developed and less-developed settings[1, 33, 34], and the presence of common STIs can enhance both HIV transmission[12, 35, 36] and progression[37]. To our knowledge, this study represents the most comprehensive assessment to date of viral and bacterial STIs in MSM in Canada, where the estimated HIV prevalence in the community exceeds 15% and HIV incidence remains high. Syphilis (current or ever) and viral STIs (including HSV-2, CMV, HPV, HBV and HCV) were very common in this clinic-based MSM population, and were consistently more frequent among men living with HIV, while urethral gonorrhea and chlamydia were relatively infrequent in all participants.

HCV is most efficiently transmitted through percutaneous route and persons who inject drugs are at greatest risk of infection. In our study sample, HCV infection was highly associated with lifetime (ever) IDU regardless of HIV infection status. The prevalence of HCV was 36.2% among IDU and 2.7% among non-IDUs, 71% of the HCV infections observed were among MSM-IDUs. Although the role of sexual transmission in HCV infection dynamics is controversial[38], a high prevalence of HCV has been observed in MSM who are infected by HIV and who do not report IDU. Among men reporting no prior IDU, the prevalence of HCV was 3.6% among MSM living with HIV and 1.4% among those living without HIV. Our results are in keeping with studies that described a higher prevalence of HCV among MSM living with HIV who lack risk factors for percutaneous exposure[30, 39, 40]. Specifically, the prevalence of HCV among MSM living with HIV and who reported no prior IDU was 3.6%, which was higher than the estimated 1.1% prevalence in the overall male population in Toronto[39]. We did not observe an association between HCV prevalence and sexual practices, perhaps because sexual behaviour during the previous 6 months differed from that at the time of HCV infection. However, prospective cohort and case-control studies have found that condomless receptive anal intercourse was linked to the risk of sexually acquired HCV infection among HIV-infected MSM[30, 41–43]. Although it can be difficult to control for additional avenues for percutaneous blood exposure during condomless sex[44], biologic evidence supports the possibility of sexual transmission, as HCV RNA has been detected in semen and other body fluids[45, 46]. The lower HCV prevalence among HIV-negative MSM who reported no IDU history (1.4%) was comparable to the general population and similar to previous studies[47, 48].

The proportion of participants with prior HBV vaccination was high in both MSM living with and without HIV, but HBV infection was not uncommon and was associated with older age and more lifetime male sexual partners, suggesting that these persons may have been infected prior to being vaccinated. Therefore, efforts should be made to reinforce timely HBV vaccination among MSM.

Our findings regarding the prevalence of ever having had syphilis are consistent with, and extend, previous findings. In particular, Burchell and colleagues[49] found that the prevalence of prior syphilis was 30.3% among Ontario MSM living with HIV in 2009; this was similar to the 36.9% observed in our study and much higher than the 5.4% prevalence they report in non-MSM men living with HIV. However, their study was unable to assess HIV-negative MSM from the same community, and the 20.4% prevalence of prior syphilis infection we observed in the latter group is still over ten-fold higher than the prevalence in the general Ontario population[49]. Oral sex is associated with transmission of syphilis. A study in Chicago, USA showed that 20% primary and secondary syphilis cases among MSM during 2000–2002 were attributed to oral sex[50]. Our study also found that syphilis was more common among MSM with condomless oral sex, suggesting that the role of oral sex in transmission should be emphasized in syphilis education programs.

While urethral gonorrhea and chlamydia were infrequent in our study, this may relate to the relatively advanced age of participants and to our lack of rectal and pharyngeal screening. Indeed, a retrospective study in San Francisco found that most asymptomatic chlamydia (77%) and gonorrhea (95%) infections would have been missed in the absence of rectal and pharyngeal screening[51]. Therefore, rectal and pharyngeal screening in MSM would be beneficial in future studies[52].

We found that the prevalence of HSV-2 (38%) and anal high-risk HPV (52%), both of which may enhance HIV susceptibility[9–11, 13], were much higher among HIV-negative MSM than previously described in the general population or other MSM communities[14, 18, 31, 32, 50, 51, 53, 54]. A study among predominantly heterosexual men from two STI clinics in Alberta, Canada found an HSV-2 prevalence of 19%[54], and the age-standardized seroprevalence of HSV-2 in the general male population aged 15 to 44 years old from Ontario, Canada was 8.6%[53]. Furthermore, a study of anal high-risk HPV infection in MSM from the United States and other countries in the region found a prevalence of 27% in MSM and 6.8% in heterosexual men[6], both considerably lower than the 51.7% that we observed in HIV-negative MSM. While HPV may impact HIV transmission, the clearest implications relate to the need for HPV vaccination and anal cancer prevention programs in men practicing receptive anal sex, where the prevalence of anal cancer and anal intraepithelial neoplasia are dramatically increased[10].

Several caveats should be noted regarding this study. The nature of this cross-sectional study does not allow us to determine whether HSV-2, HPV, HBV and HCV infection were acquired before or after HIV infection, or to infer causality in the co-infection relationships that we observed. Sexual behaviours were assessed through self-report, and there may be inaccuracies due to recall bias. Also, sexual behaviours collected in the previous 6 months of the survey may not represent those at time of HIV acquisition, particularly for persistent pathogens such as herpesvirus. While prior HBV infection was common among persons who reported HBV vaccination, our study design cannot determine whether this occurred before vaccination or represented vaccine failure.

Our study recruitment was clinic-based, and so it not clear to what extent these results can be extrapolated to MSM from Toronto who are not in medical care. The Maple Leaf Medical Clinic provides care for over 10,000 MSM, and as such is the largest provider of MSM care in Toronto. However, it is likely that men in regular care at this site differ in several ways to those not in care, or in care at less community-focused clinics. Further selection bias may also apply to participant uptake within the clinic, such that men who participated may have differed from non-participants in unknown ways. We do not believe that these potential biases would lead to an over-estimation of STI prevalence among participants, since testing and treatment for most study STIs is already available free of charge within the clinic (exceptions being screening for asymptomatic HSV1/2 and HPV); if anything, we suspect that the STI prevalence among sexually active MSM from the community who are not in care would be higher than those that we observed. Formal data regarding participation rates were not collected, but clinic staff informally estimated participation to have been approximately 95% among HIV+ men and 70% among HIV-uninfected men [Loutfy M, personal communication]. This differential participation was due in part to greater familiarity of infected men with research, as the clinic participates in numerous HIV clinical trials.

In summary, we found that several sexually transmitted co-infections were very common among MSM in Toronto living both with and without HIV, particularly HPV, several herpesviruses, viral hepatitis and syphilis. The association of these STIs with enhanced HIV transmission and with adverse health outcomes in their own right emphasizes the critical need for a community-specific prevention strategy. Enhanced STI screening, HBV and HPV vaccine-based prevention and education programs need to be implemented in this high-risk community. In addition, novel strategies currently under study in MSM communities may be useful in the future, including presumptive STI treatment and/or models of self-administered rectal and pharyngeal screening that minimize the need for health care encounters.
